# The Differential Expression of the JAK/STAT Pathway in Breast Cancer Cells Transfected with Human Papillomavirus Oncogenes

**DOI:** 10.3390/v17070880

**Published:** 2025-06-23

**Authors:** Stephanie Loureiro Leão, Gabriel Rômulo Parente da Silva, Daffany Luana dos Santos, Bianca de França São Marcos, Pedro Henrique Bezerra Fontes, Beatriz Eda de Oliveira Isídio, Isabelle Silva Simões, Elisa Fotin Genn Barros, David Beltrán Lussón, Joelson Germano Crispim, Lígia Rosa Sales Leal, Anna Jéssica Duarte Silva, Vanessa Emanuelle Pereira Santos, Antonio Carlos de Freitas

**Affiliations:** 1 Laboratory of Molecular Studies and Experimental Therapy, Department of Genetics, Federal University of Pernambuco, Av. Prof. Moraes Rego, 1235. Cidade Universitária Recife, Recife 50670-901, Pernambuco, Brazil; stephanie.lleao@ufpe.br (S.L.L.); gabriel.romulo@ufpe.br (G.R.P.d.S.); daffany.luana@ufpe.br (D.L.d.S.); bianca.saomarcos@ufpe.br (B.d.F.S.M.); pedro.bezerrafontes@ufpe.br (P.H.B.F.); beatriz.eda@ufpe.br (B.E.d.O.I.); isabelle.simoes@ufpe.br (I.S.S.); elisa.barros@estudante.fps.edu.br (E.F.G.B.); david.beltran@ufpe.br (D.B.L.); ligia.leal@ufpe.br (L.R.S.L.); anna.jessica@ufpe.br (A.J.D.S.); vanessa.emanuelle@ufpe.br (V.E.P.S.); 2 Laboratory for Immunomodulation and New Therapeutic Approaches, Research Center for Therapeutic Innovation, Federal University of Pernambuco, Recife 50670-901, Pernambuco, Brazil; joelson.crispim@ufpe.br

**Keywords:** HPV, tumor microenvironment, mammary carcinogenesis

## Abstract

Breast cancer is among the most prevalent and deadly types of cancer worldwide. Viral infections have been investigated as contributing factors in breast carcinogenesis, including infections by high-risk genotypes of human papillomavirus (HPV). Although viral DNA has been detected in breast tumors, the role of HPV activity in this type of cancer remains poorly understood. HPV oncogenes interact with various host genes, including those involved in the JAK/STAT signaling pathway. This pathway is associated with the regulation of gene expression related to the tumor microenvironment, and understanding how HPV oncogenes interact with JAK/STAT components may provide insights into the relationship between the virus and breast cancer development. In this study, we assessed the differential expression of the JAK/STAT pathway in MDA-MB-231 cells individually transfected with the E5, E6, and E7 oncogenes of HPV16. The results revealed downregulation of STAT4 in the presence of the E5, E6, and E7 oncogenes. Notably, cells transfected with E5 alone exhibited upregulation of JAK2, STAT3, and STAT6, whereas transfection with E6 and E7 resulted in their downregulation. These findings highlight the underexplored role of the E5 oncogene in contrast to the more extensively studied E6 and E7. Our results support the hypothesis that HPV oncogenes actively modulate the expression of genes involved in the tumor microenvironment in breast cancer.

## 1. Introduction

Breast cancer is one of the most common types of cancer among women, accounting for approximately 25.4% of all cases [1]. In 2020, 2.3 million cases were registered, and breast cancer resulted in 685,000 deaths around the world [1]. Breast cancer can be molecularly classified into luminal A, luminal B, HER2-positive, and triple-negative subtypes, with the latter being associated with the poorest prognoses [2,3,4]. Several risk factors are linked to the development of this cancer, including female sex, advanced age, and hormonal influences [5]. Additionally, the detection of oncogenic viral DNA in breast carcinoma tissues—including high-risk human papillomavirus (HPV) genotypes—has raised the hypothesis that such infections may be involved in breast carcinogenesis [6,7,8,9].

HPV is a widely distributed virus with multiple genotypes classified as either high-risk or low-risk based on their oncogenic potential [10]. HPV-related carcinogenesis is primarily driven by the activity of its E5, E6, and E7 oncogenes, which can promote immune modulation, tumor progression, and malignant cell survival [10]. Among the high-risk HPV types, HPV16 is the most prevalent worldwide and is strongly associated with the development of several cancers, particularly cervical cancer [11]. In breast cancer, HPV has been detected in up to 86% of analyzed samples; however, its biological role in this cancer remains unclear [12,13].

The tumor microenvironment (TME) is a dynamic, heterogeneous, and complex structure composed of various cell types that interact to influence tumor growth, immune evasion, and metastatic progression. It is modulated by factors such as hypoxia, inflammation, and cytokine signaling [14,15]. HPV can alter the TME by promoting immunosuppression, viral persistence, and disorganized proliferation, thereby contributing to carcinogenesis [16,17]. Several molecular pathways are involved in TME modulation, particularly through the regulation of gene transcription associated with these processes.

The JAK/STAT signaling pathway is one of the most important pathways in the context of the TME and is considered fundamental to cancer progression [18]. This pathway consists of Janus kinases (JAKs) and Signal Transducers and Activators of Transcription (STATs), which play a central role in immune responses and tumor development [19]. The JAK/STAT pathway is critically involved in cross-talk with other cellular pathways within the breast cancer TME—especially in triple-negative breast cancer—facilitating immune evasion and malignant cell proliferation [20].

Previously, we observed in an in vitro model that HPV oncogenes can induce immunomodulation [21]; here, using the same model, we analyzed whether HPV oncogenes can alter the expression of the JAK/STAT pathway, one of the main pathways associated with the tumor microenvironment (TME). Understanding the transcriptional modulation of these genes in the presence of HPV oncogenes provides an opportunity to explore how the virus may influence the breast cancer TME.

## 2. Materials and Methods

### 2.1. Vector Construction, Cell Culture, and Transfection

**Figure 1:**
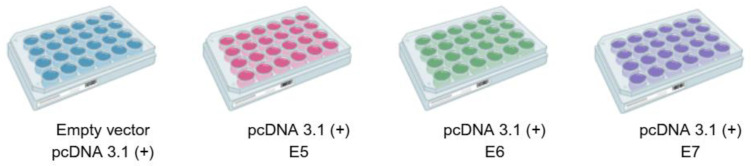
Transfection groups: MDA cultivated with pcDNA 3.1 (empty vector); transfected MDA with oncogene E5; transfected MDA with oncogene E6; transfected MDA with oncogene E7.

Vector construction, cloning, and cell transfection were carried out following the methodology previously described by Santos et al. (2024) [21]. Briefly, the E5, E6, and E7 oncogene sequences of HPV16 (GenBank accession No. K02718.1) were cloned into the pGEM-T vector and subsequently subcloned into the mammalian expression vector pcDNA3.1 (+). Following confirmation of successful cloning, plasmid DNA was isolated using a Qiagen Plasmid Plus Maxi Kit and individually transfected into MDA-MB-231 cells. This cell line is a human epithelial breast cancer line derived from triple-negative metastatic mammary adenocarcinoma. The experiment consisted of four groups, as illustrated in Figure 1.

### 2.2. RNA Extraction, cDNA Synthesis, and JAK/STAT Expression Analysis

Total RNA from transfected cells, including those expressing HPV oncogenes and those transfected with the empty vector, was extracted using a PureLink RNA Mini Kit (Invitrogen^®^, Carlsbad, CA, USA). The quality and concentration of the RNA were assessed via agarose gel electrophoresis and spectrophotometry using a NanoDrop instrument (Thermo Scientific^®^, Waltham, MA, USA), based on the 260/280 nm absorbance ratio. cDNA synthesis was performed using a Maxima First Strand cDNA Synthesis Kit with dsDNase (Thermo Scientific^®^) according to the manufacturer’s instructions. The expression levels of the transcription factors STAT4, STAT6, STAT3, and JAK2 tyrosine kinase—which are associated with the tumor microenvironment—were evaluated using a CFX Opus 96 Real-Time PCR System (Bio-Rad, Berkeley, CA, USA) with gene-specific primers (Table 1), following the MIQE guidelines [22]. Reactions were conducted using SYBR Green with GoTaq qPCR Master Mix, and GAPDH and eukaryotic translation elongation factor 1 alpha 1 (EEF1A1) were used as reference genes to normalize the relative gene expression (Table 1). All reactions were performed with five biological replicates and technical duplicates to ensure the results had greater reliability.

### 2.3. Statistical Analysis

The Kolmogorov–Smirnov test was used to assess the distribution of the gene expression data. All samples exhibited a normal distribution and were subsequently analyzed using a two-way ANOVA with a full factorial model. Relative expression analysis was performed using the 2^−ΔΔCt^ method, with the results transformed into log_2_ (fold change) values [27]. Multiple comparisons between the experimental groups and genes were conducted with significance adjustment using the Sidak correction method. Hypothesis testing was performed considering a significance level of *p* < 0.05. All statistical analyses were conducted using GraphPad Prism, version 10.3.1 (GraphPad Software, Inc., San Diego, CA, USA).

## 3. Results

### 3.1. Modulation of Gene Expression of JAK/STAT in Presence of HPV E5 Oncogene

This study built upon experiments conducted by Santos et al. (2024) [21], which demonstrated the effects of immunomodulation following transfection of HPV E5, E6, and E7 oncogenes in MDA-MB-231 cells. The expression of the E5, E6, and E7 oncogenes was confirmed by real-time PCR (RT-qPCR), and the quantification cycle (Qc) values are reported in Table 2. Further details are described by Santos et al. [21].

**Figure 2:**
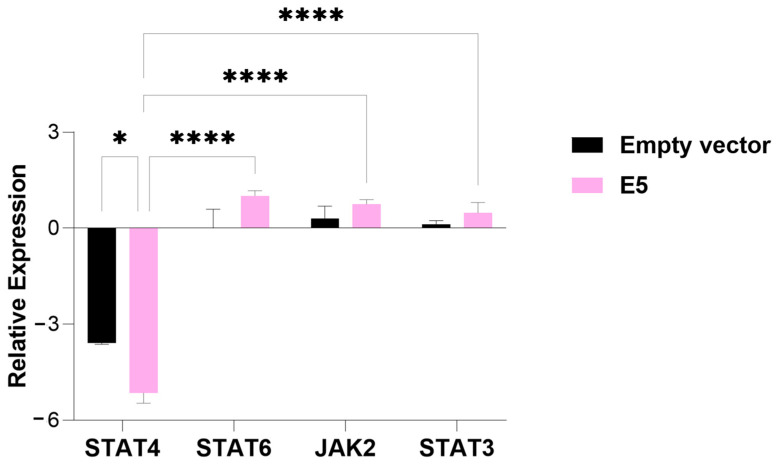
An analysis of the relative expression of the transcription factors STAT4, STAT6, STAT3, and tyrosine kinase JAK2 in MDA-MB-231 cells transfected with the E5 gene. The data represents the medians ± the pattern error of the log_2_ (fold change) of the gene expression obtained by RT-qPCR, calculated by the 2^−ΔΔCt^ method. Differences were evaluated between the groups (E5 vs. the empty vector) and between the genes inside each group. *p* < 0.05 (*); *p* < 0.00001 (****).

In cells transfected with E5, a significant decrease in STAT4 expression was observed compared to cells transfected with the empty vector (*p*-value = 0.05). Additionally, while the relative expression of STAT4 was suppressed, the transcription factors STAT6 and STAT3 and the tyrosine kinase JAK2 showed induced expression (Figure 2).

### 3.2. Modulation of Gene Expression of JAK/STAT in Presence of HPV E6 Oncogene

**Figure 3:**
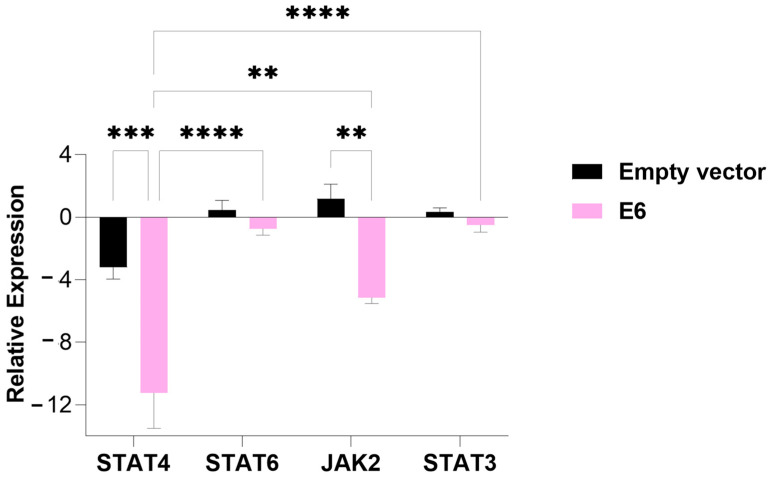
An analysis of the relative expression of the transcription factors STAT4, STAT6, STAT3, and tyrosine kinase JAK2 in MDA-MB-231 cells transfected with the E6 gene. The data represents the medians ± the pattern error of the log_2_ (fold change) of the gene expression obtained by RT-qPCR, calculated by the 2^−ΔΔCt^ method. Differences were evaluated between the groups (E6 vs. the empty vector) and between the genes inside each group. *p* < 0.01 (**); *p* < 0.0001 (***); *p* < 0.00001 (****).

In cells transfected with the E6 oncogene, the expression of all evaluated genes was suppressed. Specifically, STAT4 expression was significantly decreased compared to cells transfected with the empty vector (*p*-value = 0.0001), as observed in the E5-transfected cells. Additionally, JAK2 expression was also significantly suppressed in relation to the empty-vector control (*p*-value = 0.01) (Figure 3).

### 3.3. Modulation of Gene Expression of JAK/STAT in Presence of HPV E7 Oncogene

**Figure 4:**
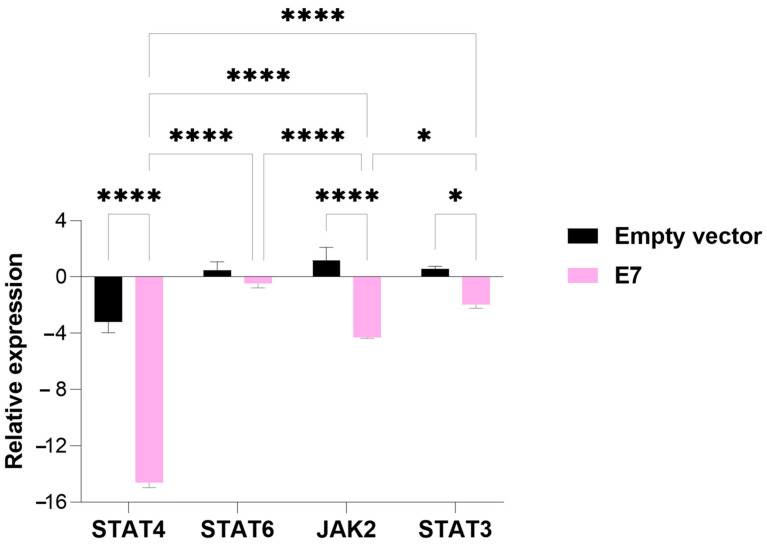
An analysis of the relative expression of the transcription factors STAT4, STAT6, STAT3, and tyrosine kinase JAK2 in MDA-MB-231 cells transfected with the E7 gene. The data represents the medians ± the pattern error of the log_2_ (fold change) of the gene expression obtained by RT-qPCR, calculated by the 2^−ΔΔCt^ method. Differences were evaluated between the groups (E7 vs. the empty vector) and between the genes inside each group. *p* < 0.05 (*); *p* < 0.00001 (****).

Similar to the E6-transfected group, the group of cells transfected with the E7 oncogene exhibited suppressed expression of all genes associated with the TME evaluated in this study. Both STAT4 and JAK2 expression were significantly reduced in cells transfected with E7 compared to those transfected with the empty vector (*p*-value = 0.00001), a result consistent with the findings for E6. Additionally, transfection with the HPV E7 oncogene also led to a significant decrease in the relative expression of STAT3 (*p*-value = 0.05) compared to the empty-vector control (Figure 4).

## 4. Discussion

HPV oncogenes are known to modulate the tumor microenvironment (TME) in cancers such as cervical and oropharyngeal cancers [28,29,30]. However, in the context of breast cancer, this remains an emerging area of study. In this study, we evaluated the impact of the presence of HPV viral oncogenes on gene expression associated with the TME, thereby providing new insights into the molecular mechanisms underlying the relationship between viral infection and mammary carcinogenesis.

The E5 viral oncogene has been associated with the regulation of growth factor receptors and the modulation of immune responses, potentially leading to decreases in these responses [31,32]. An in vitro study investigating the effects of E5 inhibition found an increase in STAT6 expression in primary human foreskin keratinocytes compared to cells with this oncogene [33]. In our study, STAT6 was the transcription factor with the highest induced expression in the presence of E5, when compared to the other genes evaluated. This transcription factor promotes the expression of genes associated with cell proliferation, such as cyclins, and inhibits pro-apoptotic genes, thereby facilitating tumor cell survival and growth [34,35].

A reduction in STAT4 activation has been linked to alterations in the cellular immune response, playing a crucial role in the regulation of Th1 lymphocytes [36]. These lymphocytes are responsible for synthesizing various cytokines, such as interferon-gamma (IFN-γ), which are essential for activating macrophages and inducing an effective immune response against tumor cells [37]. Thus, the decrease in STAT4 expression mediated by the E5 oncogene, as observed in our study, may impair Th1 lymphocyte production, weakening the immune system’s ability to identify and eliminate malignant cells, thereby contributing to immune evasion. In triple-negative breast cancers, there is typically more infiltration of lymphocytes into the tumor microenvironment. However, as seen in our results, the presence of the E5 oncogene may induce a protumoral profile in these recruited lymphocytes, facilitating cancer progression [38].

The E6 oncogene is known to deregulate the cell cycle and promote genomic instability [39]. In the present study, the presence of the E6 oncogene significantly reduced the expression of the transcription factors STAT4 and JAK2. JAK2 activation occurs when cytokines, such as IL-12, bind to their receptor, triggering its phosphorylation and activating STAT family factors, including STAT4. This activation regulates the expression of genes involved in T-cell proliferation and the immune response [40,41].

A study conducted with HPV-positive cervical cancer samples revealed that inhibition of JAK2 mediated by miR-204-5p present in E6+ exosomes, extracellular vesicles secreted by HPV-infected cells containing the E6 oncogene, promoted macrophage polarization toward the M2 phenotype, contributing to the progression of HPV-associated cervical lesions [42]. It has been shown that JAK2 mRNA expression is associated with favorable outcomes in breast cancer, particularly in recurrence-free survival, with a more pronounced protective effect in specific subtypes, such as triple-negative breast cancer [43]. The decrease in JAK2 observed in this study may impair the antitumor immune response, favoring disease progression, especially in more aggressive subtypes, such as triple-negative breast cancer [44].

STAT4 is a key transcription factor in the differentiation of CD4+ T cells into Th1 cells, a process mediated by IL-12-induced JAK2 activation. It plays a crucial role in the antitumor immune response by promoting IFN-γ production and NK cell activation. Additionally, STAT4 upregulates PD-L1 and MHC-II expression, influencing the immune response in patients [45]. A study by Gooch, Christy, and Yee (2002) [46] demonstrated that reduced STAT4 activation compromises the ability of IL-4 to induce apoptosis in breast cancer cells, thus favoring the survival and progression of malignant cells. In the present study, the presence of the HPV16 E6 oncogene reduced the expression of JAK2 and STAT4, which are key regulators of the immune response and cell proliferation, potentially negatively impacting breast cancer prognosis.

In this study, expression of the E7 oncogene in triple-negative breast cancer cells led to a decrease in the expression of the STAT4, JAK2, and STAT3 genes, which are key mediators of the immune response [47]. The viral E7 oncogene may affect the STAT4 signaling pathway by downregulating IL-6 expression and inhibiting STAT4 phosphorylation and T-box transcription factor 21 (T-bet) expression in CD8+ T cells, thereby impairing the effector function of these cells and facilitating immune evasion [48]. Data analyses from The Cancer Genome Atlas (TCGA) indicate that high expression of the IL-12/STAT4 axis is associated with better survival rates in breast cancer patients, especially in more aggressive subtypes, while reduced STAT4 expression correlates with poorer prognoses [49]. It has been shown that the HPV16 E7 oncoprotein disrupts JAK1/JAK2 signaling by suppressing IFN-γ-induced STAT1 phosphorylation, which blocks IRF-1 and TAP-1 expression [50]. This mechanism reduces the efficiency of antigen presentation via MHC class I, allowing HPV-infected cells to escape recognition by cytotoxic T lymphocytes (CTLs), facilitating immune evasion and viral persistence [50]. In our study, we observed that HPV16 E7 expression in triple-negative breast cancer cells led to a significant reduction in the expression of JAK2 and STAT4, central components of cytokine-mediated immune response pathways. Given that JAK2 and STAT4 are aligned with effective CTL activation and better clinical outcomes, their suppression by E7 suggests a potential mechanism of viral immune evasion that may not only support HPV persistence but may also contribute to an immunosuppressive environment, facilitating tumor progression [45,51].

Our results also revealed an E7-induced reduction in STAT3 expression. STAT3 is a key transcription factor in inflammatory and tumor signaling, and its dysregulation may contribute to tumor progression by impairing effective immune responses [52,53]. This negative modulation may result in decreased IL-6 expression, which directly impacts the balance of the tumor microenvironment, as IL-6 plays an essential role in immune regulation and tumor cell behavior [54,55]. A study by Kettner et al. (2019) showed that reduced IL-6 levels, in turn, promote an increase in estrogen receptor (ER) expression, a factor known to drive cell proliferation in specific breast cancer types [56]. The observed decrease in STAT3 expression in E7-transfected cells in our study demonstrates the multifaceted action of this viral protein, which not only promotes immune evasion but may also support tumor growth. This relationship highlights the relevance of STAT3 and IL-6 as potential therapeutic targets, suggesting that strategies aimed at modulating this pathway could be promising for combating tumor progression [51].

The results obtained in this study showed that STAT6 expression was not modulated by the transfected oncogenes in the MDA-MB-231 cell line. In breast cancer, STAT6 plays a key role in inhibiting growth and inducing apoptosis, as it is activated by IL-4 independent of IRS-1 [46]. It has been proposed that STAT6, acting as a transducer and activator of IFN-γ- and TNF-α-mediated signaling pathways, contributes to the regulation of cell viability in cervical cancer and improved survival, as silencing this transcription factor induces apoptosis and p53 expression [57]. The literature indicates that STAT6 plays important roles in regulating cell viability and inducing apoptosis in various cancer types [58]. However, discrepancies between our results and previous studies suggest that the interactions between HPV oncogenes and STAT6 signaling may be more complex and variable, depending on the cell type, viral oncogene, tumor context, and viral load.

In summary, the results of the present study reveal that the HPV viral oncogenes E5, E6, and E7 exert distinct effects on the expression of genes involved in the immune response and modulation of the tumor microenvironment in triple-negative breast cancer cells. As observed, all three oncogenes, E5, E6, and E7, were associated with decreased STAT4 expression, which may compromise the effectiveness of the antitumor immune response. Reduced STAT4 expression may, therefore, impair the activation of pro-inflammatory pathways, favoring a tumor microenvironment conducive to disease progression. Although most of the literature has focused on the effects of the E6 and E7 oncoproteins, our data reveal that E5 activity is a modulator of gene expression associated with the tumor microenvironment. These findings support the hypothesis that HPV, through its oncoproteins, may act in a multifaceted manner in breast cancer by affecting immune and protumor pathways.

## 5. Conclusions

The results of this study highlight the significant impact of the HPV16 oncogenes E5, E6, and E7 on the modulation of gene expression of transcription factors associated with the JAK/STAT signaling pathway in MDA-MB-231 cells, a triple-negative breast cancer cell line. The presence of the E5 oncogene in these cells led to a reduction in STAT4 expression, while the E6 oncogene primarily suppressed the expression of JAK2 and STAT3. The E7 oncogene reduced the expression of STAT4, JAK2, and STAT3. These alterations suggest a role for these oncogenes in immune evasion and tumor progression in triple-negative breast cancer. Given the critical role of the JAK/STAT pathway in the tumor microenvironment, these findings underscore the need for further investigations to elucidate the molecular mechanisms involved and evaluate the potential of these alterations as therapeutic targets or prognostic biomarkers in this aggressive subtype of breast cancer.

## Figures and Tables

**Table 1:** Primers used for amplification of STAT3, STAT4, STAT6, and JAK2 genes in RT-qPCR. GAPDH and EEF1A1 were used as reference genes for normalization of gene expression analysis. T.a—primer annealing temperature.

Gene	Sequence	T.a	Reference
EEF1A1 F	GTTGCGGTGGGTGTCATCA	60 °C	Leitão et al. (2014) [23]
EEF1A1 R	GAGTGGGGTGGCAGGTATT	60 °C	Leitão et al. (2014) [23]
GAPDH F	GAAGGTGGGGCTCATTTG	60 °C	Leitão et al. (2014) [23]
GAPDH R	TTAAAAGCAGCCCTGGTG	60 °C	Leitão et al. (2014) [23]
STAT4 F	CCTGGGTGGACCAATCTGAA	60 °C	Usui et al. (2003) [24]
STAT4 R	CTCGCAGGATGTCAGCGAA	60 °C	Usui et al. (2003) [24]
JAK2 F	TCTGGGGAGTATGTTGCAGAA	60 °C	Park et al. (2019) [25]
JAK2 R	AGACATGGTTGGGTGGATACC	60 °C	Park et al. (2019) [25]
STAT6 F	CAAAGCCCTAGTGCTGAAGAG	60 °C	Park et al. (2019) [25]
STAT6 R	CTCCTGCTGTAGCTGGGAATA	60 °C	Park et al. (2019) [25]
STAT 3 F	GGAGGAGGCATTCGGAAAG	60 °C	Zhao et al. (2018) [26]
STAT3 R	TCGTTGGTGTCACACACAGAT	60 °C	Zhao et al. (2018) [26]

**Table 2:** Confirmation of the expression of HPV16 oncogenes E5, E6, and E7 by real-time PCR. These oncogenes were individually transfected into MDA-MB-231 cells.

Gene	Quantification Cycle (Qc)
E5	25
E6	22
E7	29

## Data Availability

For access to the information provided in this study, please contact the corresponding author upon request.
